# Laparoscopic isolated caudate lobe resection

**DOI:** 10.1038/s41598-021-82262-9

**Published:** 2021-02-22

**Authors:** Maulik Parikh, Ho-Seong Han, Jai Young Cho, Mizelle D’Silva

**Affiliations:** 1 Shreemay Gastrosurgical Hospital, “Sameep”, Plot No 98, Kalubha Road, Kalanala, Bhavnagar, Gujarat 364002 India; 2 Department of Surgery, Seoul National University Bundang Hospital, Seoul National University College of Medicine, Gumi-ro, 173, Bundang-gu, Seongnam-si, Gyeonggi-do, 13620 Republic of Korea

**Keywords:** Hepatology, Surgical oncology

## Abstract

Previously, isolated caudate lobectomy was rarely performed and the caudate lobe was usually resected along with other segments. Isolated caudate lobe resection is a challenging procedure even for an experienced surgeon. Our aim was to evaluate the feasibility, safety and outcomes of laparoscopic isolated caudate lobectomy and to compare these with the open technique. We retrospectively analyzed 21 patients who underwent isolated caudate lobectomy between January 2005 and December 2018 at Seoul National University Bundang Hospital. Patients who underwent either anatomical or non-anatomical resection of the caudate lobe were included. Patients were divided into two groups according to whether they underwent laparoscopic or open surgery. Intra-operative and postoperative outcomes were compared with a median follow-up of 43 months (4–149). A total of 21 patients were included in the study. Of these, 12 (57.14%) underwent laparoscopic and nine (42.85%) underwent open caudate lobectomy. Median operation time (204.5 vs. 200 minutes, *p* = 0.397), estimated blood loss (250 vs. 400 ml, p = 0.214) and hospital stay (4 vs. 7 days, *p* = 0.298) were comparable between laparoscopy and open group. The overall post operative complication rate was similar in both groups (*p* = 0.375). The 5-year disease free survival rate (42.9% vs 60.0%, *p* = 0.700) and the 5-year overall survival rate (76.2% vs 64.8%, *p* = 0.145) was similar between laparoscopy and open group. Our findings demonstrate that with increasing surgical expertise and technological advances, laparoscopic isolated caudate lobectomy can become a feasible and safe in selected patients.

## Introduction

Over the past two decades, patients undergoing liver surgeries have experienced remarkable improvements in terms of morbidity, mortality, and long-term survival. This is partly due to improved ability to identify patients suitable for curative resections following advances in investigative procedures. Developments in surgical technology, introduction of new instruments, and low central venous pressure surgery have all contributed to the favorable outcomes of patients undergoing hepatic surgery^1^.

**Figure 1:**
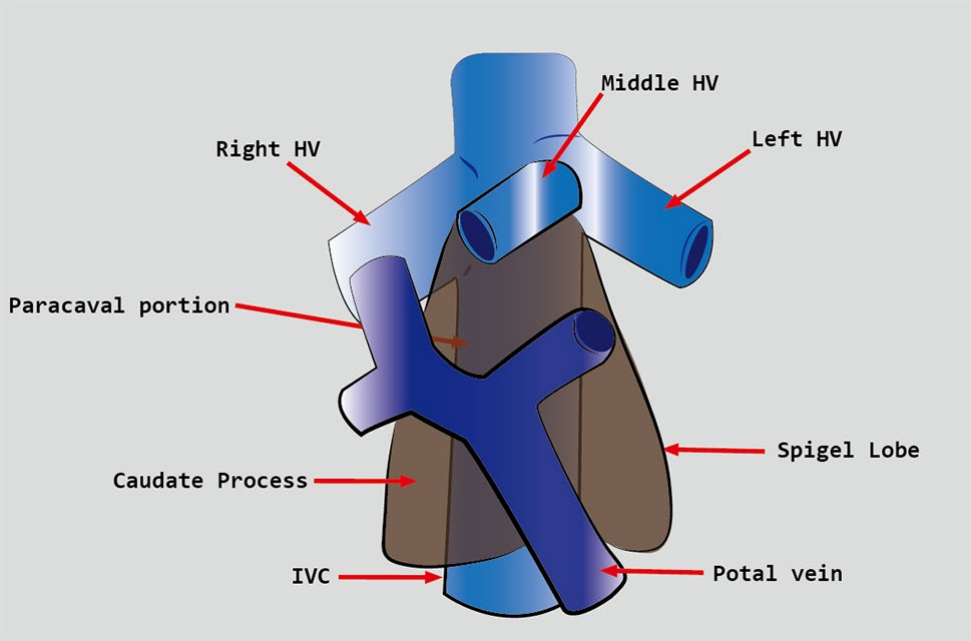
Anatomy of the caudate lobe. *HV* hepatic vein, *PV* portal vein, *IVC* inferior vena cava.

For many years, isolated caudate lobectomy was rarely performed because surgeons preferred to resect the caudate lobe together with other liver segments. This is because isolated caudate lobectomy is technically challenging, even for experienced surgeons. The difficulty of this procedure is related to the deep, complex anatomic location of the caudate lobe and its proximity to major vessels, including the inferior vena cava (IVC), the ligamentum venosum, the middle and Right hepatic vein, and the left portal vein^2^ (Fig. 1). More recently, isolated caudate lobectomy is being increasingly performed because the caudate lobe is often the only site of involvement by metastases and hepatocellular carcinoma (HCC). Isolated caudate lobectomy offers the possibility of radical resection and allows the surgeon to preserve the functional hepatic parenchyma^3^. There are four main surgical approaches for open caudate lobectomy: left-sided, right-sided, combined left- and right-sided, and anterior transhepatic^4^. A fifth approach, the retrograde approach, was recently proposed for tumors invading the IVC^5^.

Since the introduction of laparoscopic liver resection by Reich in 1991, this procedure has gained popularity worldwide^6^, and its safety and feasibility are now widely accepted. With advances in technology and increasing experience, its application has extended to lesions located in the posterior and superior segments of the liver, and to major liver resection. However, laparoscopic resection of the caudate lobe is still challenging owing to the factors described above^7^.

Currently, there is limited data comparing the outcomes of laparoscopic and open caudate lobectomy, primarily because isolated caudate lobectomy is rarely performed and is technically demanding. In this present study, we analyzed and compared the short- and long-term outcomes between laparoscopic and open caudate lobectomy.

## Methods

The study was approved by the institutional review board of Seoul National University Bundang Hospital (B-2020-6081) We conduct our studies in compliance with recognized international standards. Informed consent was waived because of the retrospective nature of the study and the analysis used anonymous clinical data. We retrospectively analyzed 21 patients who underwent isolated caudate lobectomy between January 2005 and December 2018. We included patients who underwent either anatomical or non-anatomical resection of the caudate lobe. Patients with all malignant indications for caudate lobectomy were included. If caudate lobectomy was combined with other liver resections, the patients were excluded from the study. Eligible patients were divided into two groups according to whether they underwent laparoscopic or open surgery. The intra operative and postoperative outcomes were compared. Postoperative complications were graded according to the Clavien–Dindo classification.

### Open procedure

We used the classical left-sided approach in all cases of open lobectomy. The abdomen was opened with a J-shaped incision. The peritoneal cavity was explored to exclude or identify any intra-abdominal metastases that might have been missed on preoperative imaging. Intra operative ultrasound was performed to confirm the characteristics of the tumor and to identify the tumor margin. The left liver was mobilized to the right by dissecting theround, falciform, left triangular, and left coronary ligaments. The lesser omentum was divided and the caudate lobe was exposed. Any fibrous extension of the tissue coursing over the IVC was divided to free the left margin of the caudate lobe. The caudate branches of the left and right portal veins and the left hepatic artery were ligated and divided, allowing the surgeon to lift the caudate lobe to the left and expose the retrohepatic veins. The veins were ligated or clipped, and divided from the left side of the IVC towards the right side. Complete mobilization was therefore performed. Parenchymal transection of the caudate lobe was performed using a combination of monopolar and bipolar electrocautery and a cavitron ultrasonic surgical aspirator (CUSA; Valleylab, Boulder, CO) taking particular caution when transecting the superior portion of the lobe, close to the middle and left hepatic veins. Any unexpected bleeding from the hepatic veins was controlled. After careful hemostasis, a fibrin glue sealant (Greenplast, Green Cross Corp., Seoul, Korea) was applied to the cut surface. A silastic drain was placed and, after thorough lavage, the abdomen was closed.

### Laparoscopic procedure (Total anatomic caudate resection)

**Figure 2:**
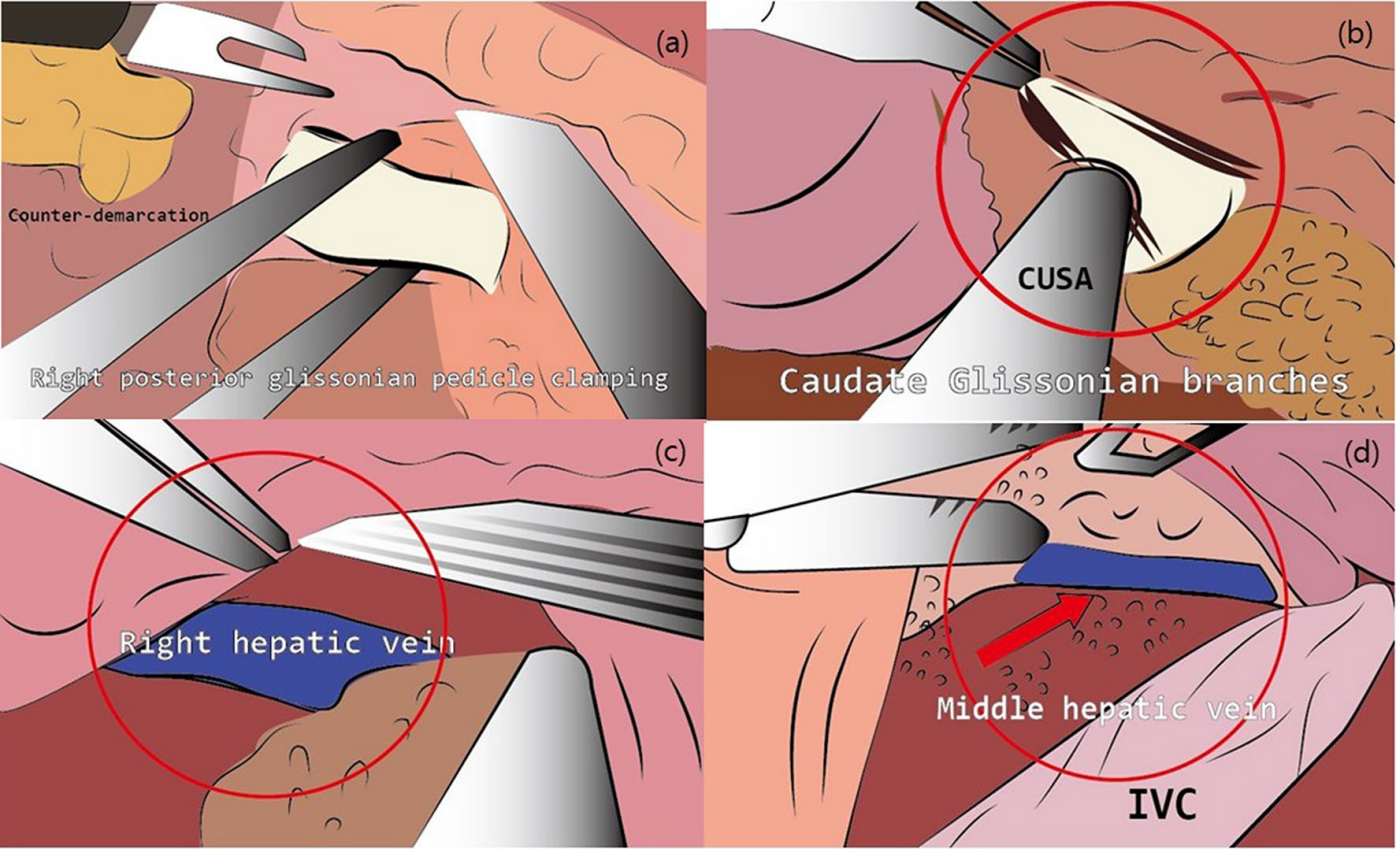
Technical aspect of laparoscopic caudate lobectomy. (**a**) The right posterior glissonian pedicle was isolated and temporarily clamped. (**b**) The caudate glissonian branches were identified and controlled. (**c**) The exposure of right hepatic vein. (**d**) After complete resection of caudate lobe, the exposure of middle hepatic vein.

**Figure 3:**
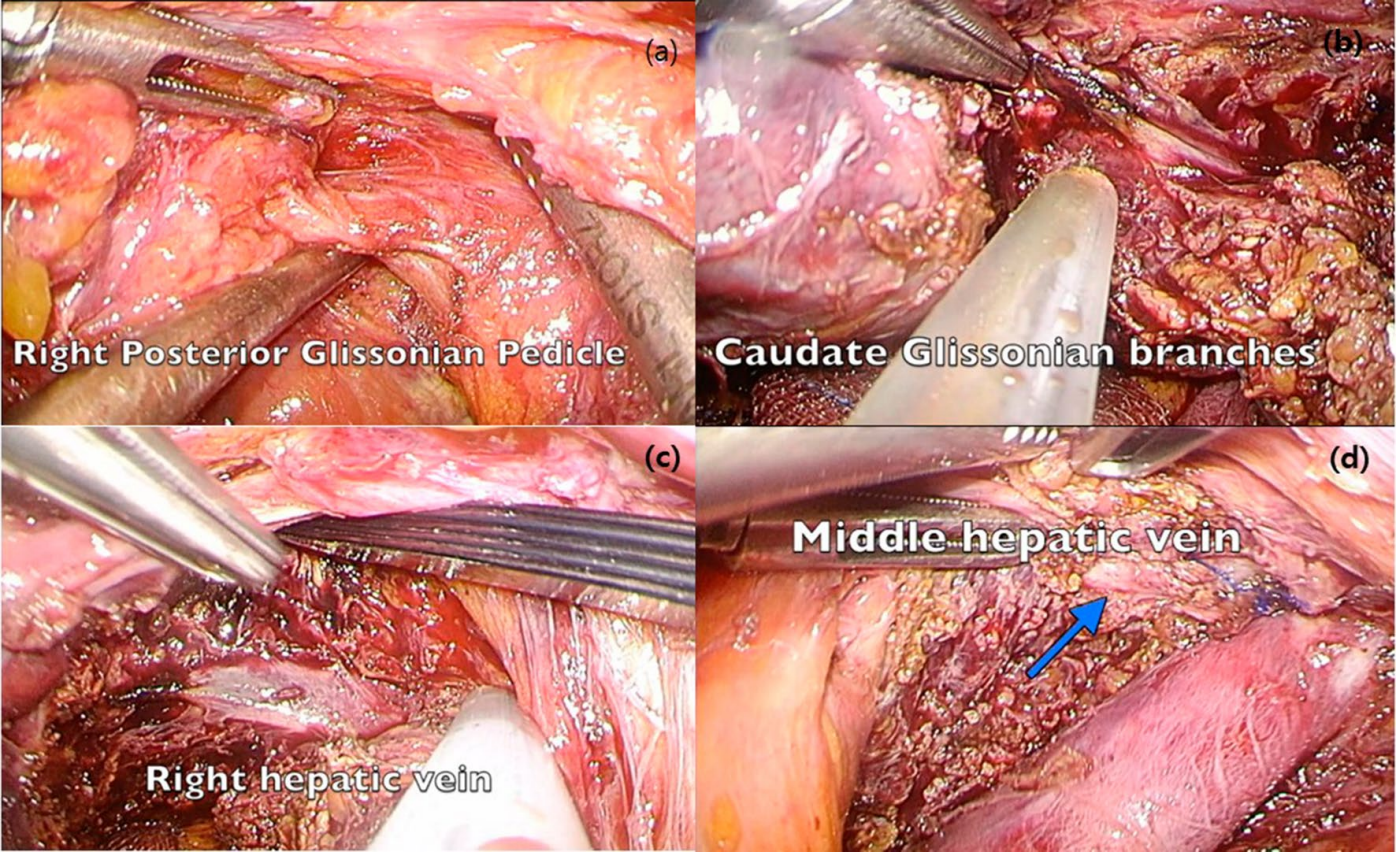
Photos taken from laparoscopic caudate lobectomy (**a**) The right posterior glissonian pedicle was isolated and temporarily clamped. (**b**) The caudate glissonian branches were identified and controlled. (**c** The exposure of right hepatic vein. (**d**) After complete resection of caudate lobe, the exposure of middle hepatic vein.

Under general anesthesia, the patient was placed in the lithotomy position. We performed five-port laparoscopy. Pneumoperitoneum was established using the infraumbilical port. Four 12-mm ports were placed under direct vision. The following steps mimicked those performed in open surgery. Intraoperative ultrasound was done to exclude undetected lesions and to visualize the relationship between the caudate lobe and the major vascular structures. The left liver was mobilized and rotated to the right. The lesser omentum was divided to expose and mobilize the caudate lobe. The fibrous attachments between the caudate lobe and the IVC were divided. The right Glissonian pedicle was isolated and temporarily clamped with a bulldog clamp. The counter demarcation of the caudate process and the right posterior section were marked with electrocautery (Figs. 2(a), 3(a)). Superficial parenchymal transection was performed with a harmonic scalpel (Ethicon Endosurgery, Inc., Cincinnati, OH) and the deeper portion was transected by laparoscopic CUSA. The posterior surface of the caudate lobe was separated from the IVC and the short hepatic veins were controlled with clips and Ligasure (Covidien, Mansfield, MA). The portal branches of the caudate lobe were cut (Figs. 2(b), 3(b)). The peripheral section of the right hepatic vein (RHV) was identified and dissection of the paracaval portion of the caudate lobe was continued along the RHV (Figs. 2(c), 3(c)). The caudate lobe was separated from the middle hepatic vein and any bleeding was controlled (Figs. 2(d), 3(d)). The short hepatic veins and the portal branches to the caudate lobe were controlled in a left-to-right fashion and the whole lobe was subsequently resected. The specimen was removed with an endobag. The cut surface was covered with fibrin glue (Greenplast). A silastic drain was placed and the ports were closed in layers.

### Statistical analyses

Data were analyzed using SPSS Software for Windows version 20 (IBM, Chicago, IL, USA). Categorical variables were tabulated as the frequency and percentage, and continuous variables as the median (interquartile range) or mean (standard deviation) after testing for normality. Survival curves for disease-free and overall survival were plotted using the Kaplan–Meier Method. A *p *value of < 0.05 was considered statistically significant.

## Results

A total of 74 patients underwent caudate lobectomy at Seoul National University Bundang Hospital between January 2005 and December 2018. Of these, 22 patients underwent isolated caudate lobectomyand were selected for this study. One patient with hemangioma of the caudate lobe was excluded. Of the remaining 21 patients, 12 (57.14%) underwent laparoscopic and nine (42.85%) underwent open caudate lobectomy.

The mean age of patients in both groups was 62 years. There were nine males in the laparoscopy group (75%) and five in the open group (55.6%). The preoperative data were comparable in both groups (Table 1).

**Table 1:** Baseline characteristics.

	Laparoscopy (N = 12)	Open (*N* = 9)	*p *value
Age, years (range)	62 (38–89)	62 (48–68)	0.364
Sex, males (%)	9 (75.0)	5 (55.6)	0.350
BMI,kg/m^2^ (range)	24.56 (20.97–30.14)	24.4 (16.49–29.88)	0.397
Hb, gm% (range)	13.95 (12.7–16.0)	12.8 (9.4–15.7)	0.391
Platelets, × 1000/μL (range)	178 (83–249)	231 (77–317)	0.397
Bilirubin, mg/dL (range)	0.65 (0.4–1.6)	1.1 (0.4–1.8)	0.230
INR (range)	1.09 (0.98–1.19)	1.07 (0.9–1.4)	0.367
ALT, IU/l (range)	74.5 (11–233)	92 (11–307)	0.460
Albumin, g/dL (range)	4 (3.2–4.5)	3.5 (2.7–3.7)	0.147
AFP, ng/mL (range)	4.4 (1.4–167)	145.4 (5.1–2605)	0.456
CEA, ng/mL (range)	3.3 (3.0–3.6)	8.2 (2.6–141.6)	0.321
Cirrhosis (%)	6 (50.0)	3 (33.3)	0.445
RFA (%)	2 (16.7)	1 (11.1)	0.798

*BMI* body mass index, *Hb* hemoglobin, *INR* international normalized ratio, *ALT* alanine aminotransferase, *AFP* α-fetoprotein, *CEA* carcinoembryonic antigen, *RFA* radiofrequency ablation.

Three out of 12 patients (25%) in the laparoscopy group underwent anatomical caudate lobe resection versus one out of nine patients (11.1%) in the open group. There was no significant difference in the mean operative time between the laparoscopy and open groups (204.5 vs. 200 min, respectively, *p* = 0.397). The estimated blood loss was similar between laparoscopy and open group (250 vs. 400 mL, respectively, *p* = 0.214). One patient in the open group required blood transfusion (*p* = 0.237). None of the patients required open conversion in the laparoscopy group. Oral diet was resumed on postoperative day 1 in 91.7% of patients in the laparoscopy group and 66.7% of patients in the open group (*p* = 0.149). The median postoperative stay was 4 days in the laparoscopy group versus 7 days in the open group (*p* = 0.298). The proportion of patients who was diagnosed with HCC (91.7% vs. 44.4%) or metastasis (8.3% vs. 44.4%) was no statistically differences between laparoscopy and open group (*p* = 0.056). Tumor size (cm, range) was almost identical in both groups (2 (0.9–4.1) vs. 2.7 (1.5–5.5), *p* = 0.299). All patients in the laparoscopy group underwent R0 resection, whereas one patient in the open group underwent R1 resection because the tumor was exposed at the resection margin. The overall postoperative complication rate was similar in both groups (16.7% vs. 33.3%, *p* = 0.375). One patient in each group experienced a major complication. In the laparoscopy group, one patient developed postoperative bile leak at 6th postoperative day. With exploration, there was a hole in left hepatic duct, which was repaired with suture and t-tube insertion. The patient was discharged without further complications. One patient in the open group developed intra-abdominal fluid accumulation that required insertion of a percutaneous drain under ultrasound guidance. Other complications in the laparoscopy group included pleural effusion, while in the open group pleural effusion and paralytic ileus (Table 2).

**Table 2:** Perioperative, postoperative, and pathological outcomes.

	Laparoscopy (*N* = 12)	Open (*N* = 9)	*p*-Value
Anatomical resection (%)	3(25%)	1(11.1%)	0.603
Operation time, min (range)	204.5 (75–450)	200 (120–550)	0.397
Blood loss, mL (range)	250 (0–650)	400 (100–1500)	0.214
Blood transfusion (%)	0 (0)	1 (11.1)	0.237
Anatomical resection (%)	3 (25)	1 (11.1)	0.422
Open conversion (%)	0 (0)	–	
Oral diet (%)			0.149
Postoperative day 1	11 (91.7)	6 (66.7)	
Later	1 (8.3)	3 (33.3)	
Hospital stay, days (range)	4 (2–10)	7 (2–27)	0.298
Diagnosis (%)			0.056
Metastases	1 (8.3)	4 (44.4)	
HCC	11 (91.7)	4 (44.4)	
Scirrhous carcinoma	0 (0)	1 (11.1)	
Edmonson–Steiner grade (%)			0.141
I	4/11 (36.3)	0 (0)	
II	6/11 (54.5)	2/4 (0.5)	
III	1/11 (9.0)	2/4 (0.5)	
IV	0 (0)	0 (0)	
pT stage (%)			0.216
I	6/11 (54.5)	2/4 (0.5)	
II	5/11 (45.4)	1/4 (0.25)	
III	0 (0)	1/4 (0.25)	
IV	0 (0)	0 (0)	
Tumor size, cm (range)	2 (0.9–4.1)	2.7 (1.5–5.5)	0.299
Resection margin, cm (range)	0.7 (0.1–2.2)	0.2 (0.0–1.0)	0.405
R status (%)			0.237
R0	12/12 (100)	8/9 (88.8)	
Complications (%)	2 (16.7)	3 (33.3)	0.375
Clavien–Dindo grade (%)			
Major Complications	1 (8.3)	1 (11.1)	0.329

**Figure 4:**
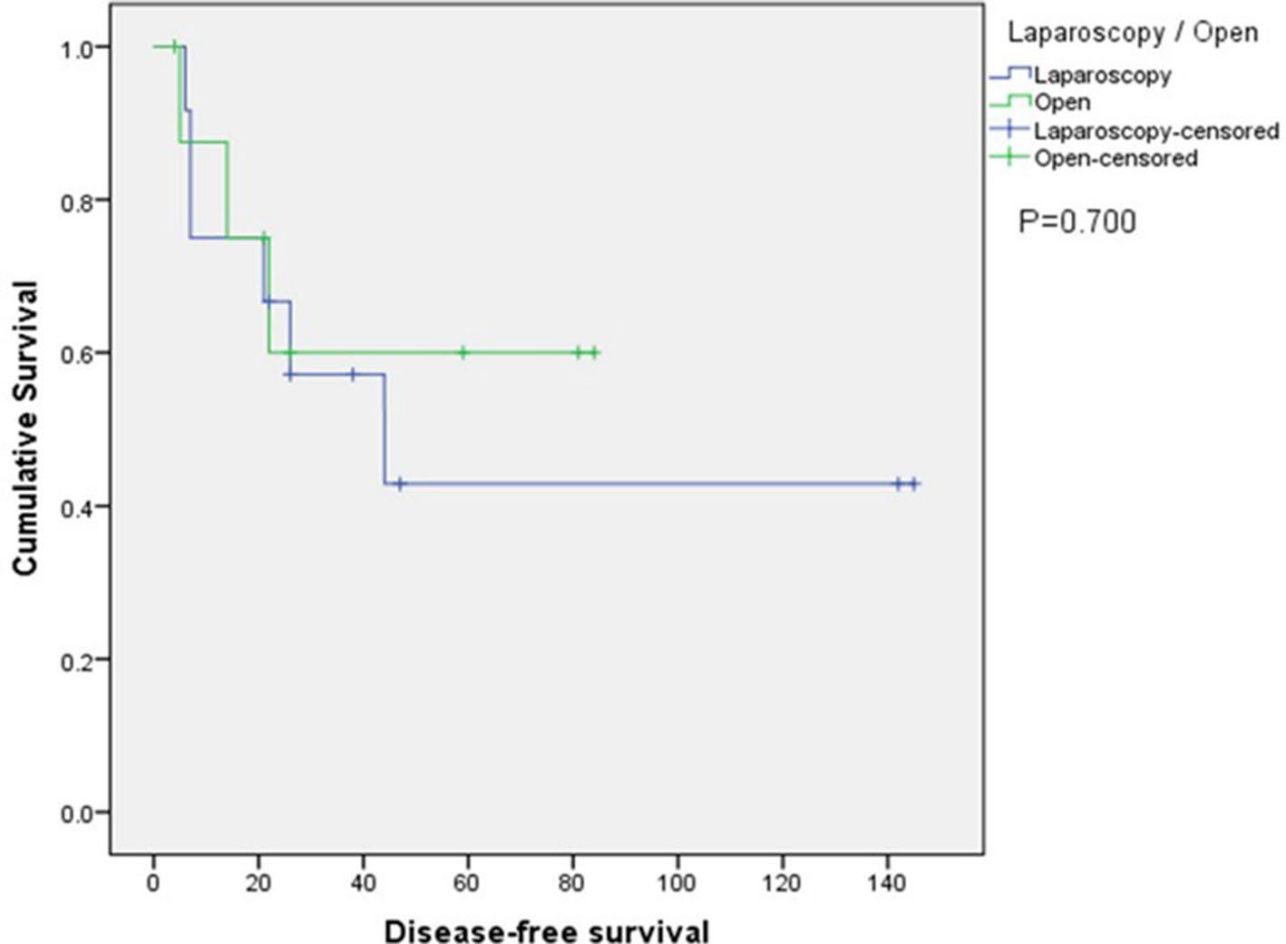
Kaplan–Meier plot of disease-free survival.

**Figure 5:**
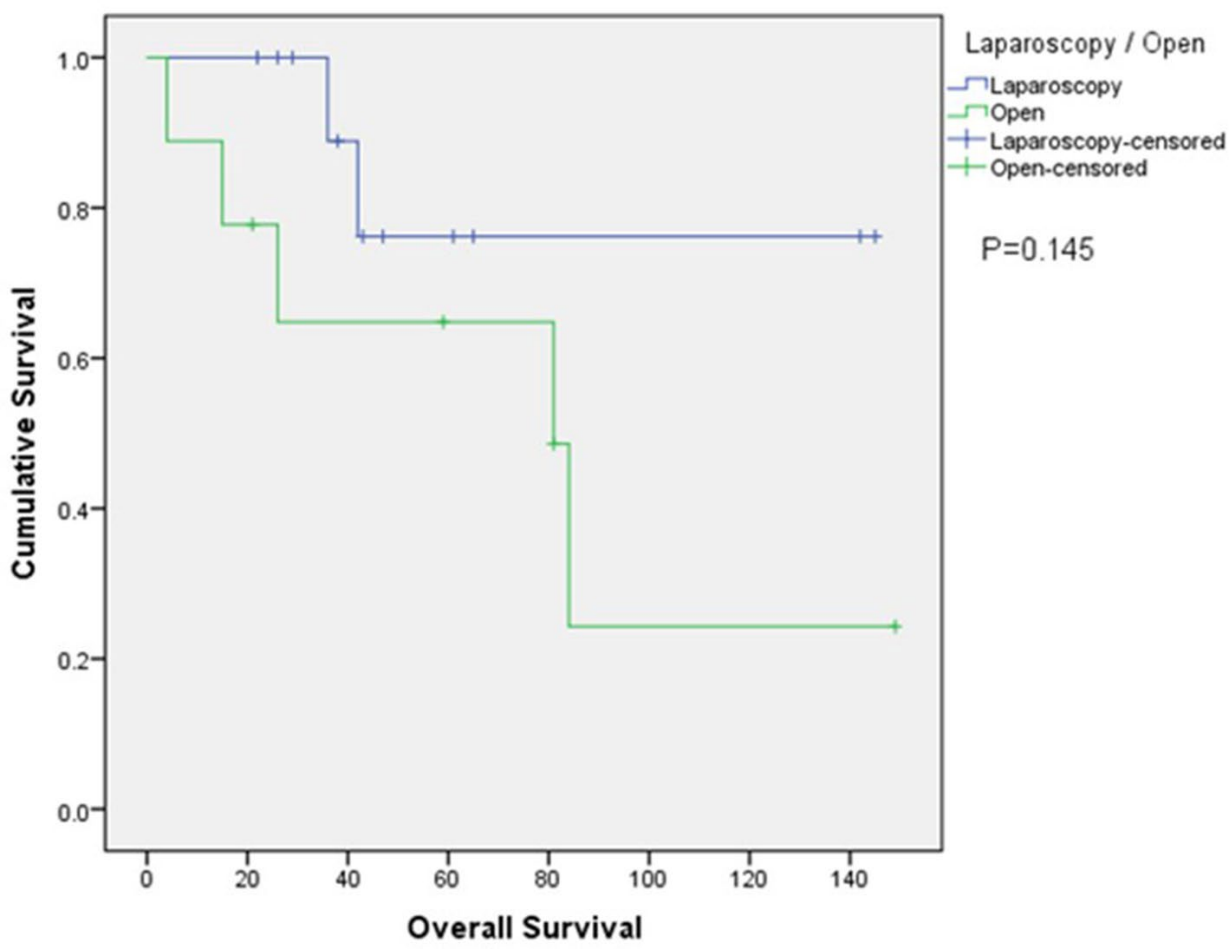
Kaplan–Meier plot of overall survival.

During a median follow-up of 43 months, the 5-year disease free survival rate (42.9% vs 60.0%, p = 0.700) (Fig. 4) and the 5-year overall survival rate (76.2% vs 64.8%, p=0.145) (Fig. 5) was similar between laparoscopy and open group.

## Discussion

The caudate lobe is the dorsal portion of the liver that embraces the retrohepatic IVC. It is a separate, autonomous, and unique lobe of the liver having its own vascularization and biliary drainage. The caudate lobe has three portions. The first is the left lobe, or Spiegel’s lobe (Couinaud’s segment 1), which is the main bulk of the caudate lobe and lies on the left side of the IVC and in the lesser omental bursa. The second is the caudate process, which is the continuation of the Spiegel lobe to the right between the left portal vein and the IVC, and fuses with the right lobe of the liver. The third, smaller portion is the paracaval portion, often referred to as Couinaud’s segment 9, and lies immediately posterior to segment IV. The unique anatomical location of the caudate lobe, deep into the hepatic parenchyma, and its close proximity to major vessels, including the IVC, middle and Right hepatic veins, portal vein and ligamentum venosum, make it very difficult for a surgeon to explore the caudate lobe during surgery^2, 7^.

There are three porta hepatis described in literature—the first porta hepatis is the hepatic hilum, second is the confluence of hepatic veins with the IVC, and third is the segment of the retrohepatic IVC with a series of short hepatic veins. The caudate lobe is surrounded by all three porta hepatis and there is a high risk of significant hemorrhage from these important structures during resection of the caudate lobe^8^. Previously, extended hemi-hepatectomy was considered to be the most suitable approach for malignant tumors located in the caudate lobe owing to its close proximity to the hepatic veins^9^. However, in the last few years, isolated caudate lobectomy has become more widespread. Moreover, isolated caudate lobectomy offers an opportunity for performing radical resection while preserving functional hepatic parenchyma^3^.

As described above, there are several approaches for open caudate lobectomy. In an improved anterior hepatic approach, which differs from the standard anterior approach, the liver is transected along the left lateral section^10^. In general, a left-sided approach is usually more suitable because it is generally easy to perform^11, 12^. We also used the left-sided approach in all patients who underwent open lobectomy.

Since the first reported case of laparoscopic wedge resection of the liver in 1991, many reports have demonstrated the safety and feasibility of laparoscopic liver resections. However, laparoscopic liver resection is performed at a small number of institutions with expertise in performing both hepatobiliary and laparoscopic surgery. There are several limitations of laparoscopic liver resection, including the loss of tactile sensation, difficulty of maintaining adequate traction, and the narrower view of the dissection plane, which may ultimately lead to increased blood loss and longer operation time compared with open resection^13^. However, advances in imaging, instruments, surgical technique, and surgical experience have led to increased uptake of laparoscopic resection^14^.The main technical challenges of caudate lobectomy are proper exposure of the caudate lobe due to its location deep inside the liver and to control bleeding, which may be substantial if the surrounding major vessels are injured. Compared with open surgery, laparoscopy provides a unique viewing angle from below with superior magnification and illumination, allowing greater visibility of this deep region that is otherwise difficult to visualize by the naked eye^15^. Bleeding from the short hepatic veins or IVC is very difficult to control, even during open surgery, as is bleeding from the dorsal aspect of the liver^6, 15^. Such bleeding is also difficult to control during laparoscopy because of the limited space available to move the scope and instruments^16^. Besides the advantage of improved visibility of the caudate lobe from below^15^, the use of CO_2_ to establish pneumoperitoneum, maintaining pneumoperitoneum at 10–12 mmHg^17^, intermittent Pringle maneuver^13^, and maintaining a lower central venous pressure can help to reduce blood loss. We observed lower blood loss in the laparoscopy group (240 mL) as compared to the open group (400 mL).

Anatomical hepatic resection is associated with better survival outcomes than non-anatomical resection^18^. Anatomical resection follows oncological principles by removing all of the liver parenchyma with potential tumor involvement^7^. Moreover, anatomical resection is generally safer with less intraoperative blood loss owing to ligation of the tumor-bearing portal pedicles with limited parenchymal resection^14^. In our study, three out of 12 patients in the laparoscopy group (25%) and one out of nine patients in the open group (11.1%) underwent anatomical resection of the caudate lobe.

Laparoscopic caudate lobectomy should be performed by surgeons with experience of performing a number of open hepatobiliary procedures to ensure they have gained sufficient knowledge of liver anatomy, particularly of the caudate lobe. Accordingly, there is a steep learning curve for surgeons considering laparoscopic liver resection. This is a process aimed at safely reducing operation time and blood loss which is continuously evolving^19^. This is what is followed in most high volume centers like ours, and hence there was no major difference in operative time between the laparoscopy (204.5 min)and the open group (200 min) in our study. This finding was similar to the multicenter study done by Xu et al.^6^.

The rate of early postoperative complications was similar in both the groups—while slightly on the lower side in the laparoscopy group. Both groups had one patient each who had a major complication (Clavien–Dindo classification grade IIIa and higher) postoperatively. In the laparoscopy group the patient had a bile duct injury, which required repeat surgery, while in the open group a percutaneous drain was inserted for a patient due to intrabdominal fluid collection.

Laparoscopic surgery has several advantages, including reduced postoperative pain and early mobilization. In our study we found that oral diet was started sooner, resulting in a shorter hospital stay in the laparoscopy group as compared to the open group. Simillis et al. evaluated eight nonrandomized studies and concluded that laparoscopic liver resection is comparable to or better than open resection in terms of intraoperative blood loss and the length of hospital stay^20^.

Besides the technical challenges faced in laparoscopic surgery, another issue is its oncological safety. In laparoscopy, the tumor cannot be palpated manually, which may result in inadequate resection. However, in our study, all patients in the laparoscopy group had R0 resection with a surgical margin of 0.7 cm compared with 0.2 cm in the open group. One patient in the open group had R1 resection because the tumor margin was exposed at the time of surgery. Better illumination and a magnified view in laparoscopy may explain the adequate margin achieved with laparoscopy^6^.

In terms of the survival outcomes in this study, the disease-free survival rate was lower in the laparoscopy group compared to the open group (42.9% vs. 60.0%, respectively). The presence of majority of HCC cases (11 out of 12) in the laparoscopy group with the inherent propensity of HCC for recurrence might account for this result. On the other hand, the 5-year overall survival rate was higher in the laparoscopy group (76.2%) compared to the open group (64.8%). However, these differences were not statistically significant. To our knowledge, our study is the first of its kind comparing long term and short term outcomes in patients undergoing caudate lobectomy.

This study has some limitations. It is a retrospective study with a small sample size. Further studies with a larger study population are required to analyze the outcomes of laparoscopic caudate lobectomy in greater detail. Since isolated caudate lobectomy is a relatively rare procedure, multicenter studies may be required in the future.

We therefore conclude, despite the technical difficulties associated with isolated caudate lobectomy, including the challenging anatomy and proximity to major vessels, with increased surgical expertise and technological advances, acceptable outcomes can be achieved with laparoscopic isolated caudate lobectomy. Our findings demonstrate that laparoscopic isolated caudate lobectomy seems to be feasible and safe in selected patients.
