# Author Correction: Laparoscopic isolated caudate lobe resection

**DOI:** 10.1038/s41598-021-94379-y

**Published:** 2021-07-14

**Authors:** Maulik Parikh, Ho-Seong Han, Jai Young Cho, Mizelle D’Silva

**Affiliations:** 1 Shreemay Gastrosurgical Hospital, “Sameep”, Plot No 98, Kalubha Road, Kalanala, Bhavnagar, Gujarat 364002 India; 2 Department of Surgery, Seoul National University Bundang Hospital, Seoul National University College of Medicine, Gumi-ro, 173, Bundang-gu, Seongnam-si, Gyeonggi-do, 13620 Republic of Korea

Correction to: *Scientific Reports* 10.1038/s41598-021-82262-9, published online 22 February 2021

The Article contains an error in Table 2, where the row ‘Anatomical resection (%)’ is duplicated. The correct Table 2 appears below.

**Table 2:** A correct version of the original Table 2.

	Laparoscopy (*N* = 12)	Open (*N* = 9)	*p*-Value
Anatomical resection (%)	3 (25%)	1 (11.1%)	0.603
Operation time, min (range)	204.5 (75–450)	200 (120–550)	0.397
Blood loss, mL (range)	250 (0–650)	400 (100–1500)	0.214
Blood transfusion (%)	0 (0)	1 (11.1)	0.237
Open conversion (%)	0 (0)	–	
Oral diet (%)			0.149
Postoperative day 1	11 (91.7)	6 (66.7)	
Later	1 (8.3)	3 (33.3)	
Hospital stay, days (range)	4 (2–10)	7 (2–27)	0.298
Diagnosis (%)			0.056
Metastases	1 (8.3)	4 (44.4)	
HCC	11 (91.7)	4 (44.4)	
Scirrhous carcinoma	0 (0)	1 (11.1)	
Edmonson–Steiner grade (%)			0.141
I	4/11 (36.3)	0 (0)	
II	6/11 (54.5)	2/4 (0.5)	
III	1/11 (9.0)	2/4 (0.5)	
IV	0 (0)	0 (0)	
pT stage (%)			0.216
I	6/11 (54.5)	2/4 (0.5)	
II	5/11 (45.4)	1/4 (0.25)	
III	0 (0)	1/4 (0.25)	
IV	0 (0)	0 (0)	
Tumor size, cm (range)	2 (0.9–4.1)	2.7 (1.5–5.5)	0.299
Resection margin, cm (range)	0.7 (0.1–2.2)	0.2 (0.0–1.0)	0.405
R status (%)			0.237
R0	12/12 (100)	8/9 (88.8)	
Complications (%)	2 (16.7)	3 (33.3)	0.375
Clavien–Dindo grade (%)			
Major complications	1 (8.3)	1 (11.1)	0.329

